# Separation of Symphysis Pubis during Parturition

**Published:** 1887-05

**Authors:** J. M. Sanders

**Affiliations:** Magnolia, S. C.


### SEPARATION OF THE SYMPHYSIS PUBIS DURING PARTURITION.

*Editors Atlanta Medical and Surgical Journal:*

In the February number of the *Archives of Gynæcology*, the subject of the separation of the symphysis pubis during parturition is once more brought prominently forward. I imagine that such cases are quite rare, having had personal knowledge of but one such in forty years. This one was clearly attributable to violent extractive force in the hands of an old, presumptuous, female accoucheur; yet she boasted of having attended 3,000 women in confinement.

If ever I had to witness scenes of consternation, woe and the "gush of human tears," it has been when I was hastily summoned to assist in and "share the responsibility" of the case of a poor creature in travail (shoulder and arm presentation, perhaps), whose systemic powers were completely exhausted by long and fruitless efforts at bearing down, exhorted so to do, and mashed and tugged at by her stupid murderer, who was utterly ignorant of the fact that she was thereby fearfully increasing the danger and the difficulties of deliverance.

Far be it from me to discountenance the practice of obstetricy by intelligent and trained women. Indeed, I think it should be the prerogative of such to serve in this capacity; but the misfortune is that many upstarts set out on this line who do not know their a, b. c's—overly zealous, without knowledge, and whose experience consists only in having blindly fumbled about in a case or so of natural labor. But I rejoice that, in this regard, a better day is dawning upon the human family. It is now seldom that a case of labor occurs without the presence of a physician, excepting among the most ignorant whites or voo-doo Negroes. Thus the mortality of childbirth, as well as the entailment of life-long suffering upon mothers, has notably diminished.

*Magnolia, S. C., March, 1887.* J. M. SANDERS, M. D.

